# Supplementary material for: Mapping the evolution of fertility support policies in China: A content and instrumental analysis
Source: PLoS One. 2025 Oct 9;20(10):e0332137. doi: 10.1371/journal.pone.0332137 (PMC12510515; doi:10.1371/journal.pone.0332137)
Supplement: S1 Appendix — (ZIP) [file pone.0332137.s001.zip › S1 Appendix. 226 original policy documents/15-国家人口计生委办公厅关于印发2012年流动人口计划生育全国“一盘棋”工作方案的通知(FBM-CLI-4-171551).doc]

国家人口计生委办公厅关于印发2012年流动人口计划生育全国“一盘棋”工作方案的通知

**发布部门：** [国家人口和计划生育委员会(含国家计划生育委员会)(已撤销)](https://www.pkulaw.com/law/chl?Aggs.IssueDepartment=60304) [机构沿革](javascript:void(0))

**发文字号：**人口办流管函〔2012〕85号

**发布日期：**2012.04.06

**实施日期：**2012.04.06

**时效性：** [现行有效](https://www.pkulaw.com/law/chl?Aggs.TimelinessDic=01)

**效力级别：** [部门工作文件](https://www.pkulaw.com/law/chl?Aggs.EffectivenessDic=XE0303)

**法规类别：** [计划生育管理](https://www.pkulaw.com/law/chl?Aggs.Category=09705)

国家人口计生委办公厅关于印发2012年流动人口计划生育全国“一盘棋”工作方案的通知
（人口办流管函〔2012〕85号）

各省、自治区、直辖市人口计生委，计划单列市、新疆生产建设兵团人口计生委，解放军、武警部队计划生育领导小组办公室，中直机关、中央国家机关人口计生委，委机关各单位，各直属、联系单位：
　　现将《2012年流动人口计划生育全国“一盘棋”工作方案》印发给你们，请结合实际，认真贯彻执行。

　　二〇一二年四月六日

　　2012年流动人口计划生育全国“一盘棋”工作方案

　　经过全国人口计生系统和相关部门三年来的不懈努力，全国范围内已基本建立起流动人口计划生育“统筹管理、服务均等、信息共享、区域协作、双向考核”的工作机制。为做好2012年流动人口计划生育工作，特制定本方案。
　　**一、**指导思想
　　深入贯彻落实《[流动人口计划生育工作条例](https://www.pkulaw.com/chl/f6b2c35d55b7a40ebdfb.html)》（以下简称[《条例》](https://www.pkulaw.com/chl/f6b2c35d55b7a40ebdfb.html)），以稳定低生育水平为目标，以落实双向管理职责为重点，按照“强基础、抓落实、促平衡”的总体要求，规范基层服务管理，加强层级监督，推进流动人口计划生育基本公共服务均等化，巩固完善全国“一盘棋”机制，全面提升流动人口计划生育服务管理水平。

　　**二、**总体目标
　　流动人口计划生育服务管理法规制度进一步健全，层级监管机制逐步建立；基本实现国家和省级流动人口计划生育服务管理信息系统的对接，信息准确率明显提高，重点对象协查信息反馈率、及时率达到90%，信息通报率、接收率达到80%；流动人口计划生育免费服务落实率达到85%以上，流动人口合法权益得到较好维护。

　　**三、**重点任务
　　（一）完善法规制度，规范基层服务管理。
　　1.强化统筹管理，推进流动人口计划生育综合治理，做好相关政策的衔接协调。落实“一把手”负责制，加强流动人口计划生育工作领导保障机制建设。
　　2.修订完善《全国流动人口计划生育服务管理工作规范》，研究制定流动人口一孩生育服务登记管理办法。认真贯彻落实[《条例》](https://www.pkulaw.com/chl/f6b2c35d55b7a40ebdfb.html)及相关法规制度，落实各方服务管理责任。
　　3.优化完善基层流动人口计划生育服务管理网络，健全适应流动人口计划生育基本公共服务需求的机构设置和人员配备，建立逐年增长的经费投入保障机制。
　　（二）提高统计调查质量，增强网络化协作的及时性、有效性。
　　1.完善国家、省级全员流动人口数据库，提高流动人口数据质量。建立流动人口数据定期清理、核查、评估制度，增强数据完整性和准确性。强化统计监测数据分析应用，组织开展统计监测分析报告评比。
　　2.扎实推进两级系统对接工作，规范省级系统数据统计、上报标准；优化PADIS流动人口子系统，完善层级监管功能，定期对省级系统省内应用和数据交换情况进行检查。
　　3.深化网络化协作，将提高协查信息反馈及时率和通报信息接收及时率作为工作重点，提高信息互通效率。依托国家、省级系统，探索流动人口婚育证明的电子化改革。
　　（三）推进服务均等，基本实现计划生育基本公共服务全覆盖。
　　1.深化流动人口计划生育基本公共服务均等化试点，做好评估，总结经验。落实国家规定的计划生育免费服务项目，扩大流动人口优生优育、优先优惠、奖励优待、关怀照顾等服务和政策的覆盖面。指导各地将流动人口纳入免费孕前优生健康检查项目覆盖人群，落实相关服务。
　　2.以计划生育技术服务机构和社区服务中心为依托，扩大流动人口计划生育服务网点，增强各项服务的易得性和可及性。以流动育龄人口为基础因素，建立健全计划生育药具经费动态调拨机制，努力实现流动人口免费计划生育药具全覆盖。
　　3.畅通维权渠道，严肃查处侵害流动人口权益的行为，及时办理和反馈信访案件，严格依法行政，维护流动人口合法权益。加强流动人口计划生育协会工作，推进流动人口诚信计生服务管理。
　　4.加强与相关部门的政策衔接，使实行计划生育的流动人口家庭优先享受流入地各项基本公共服务。深入开展“关怀关爱”系列活动，帮扶、救助流动人口计划生育困难家庭。
　　（四）健全层级监管制度，落实双向服务管理职责。
　　1.加强省际间政策协调，建立逐级监管投诉、协调制度。健全流动人口计划生育双向考核评估指标体系和分类考核评估制度，组织开展流动人口计划生育服务管理工作专项检查。
　　2.加强泛长三角、泛珠三角、环渤海和西北四大协作区的沟通交流和协作机制建设，加强研究解决区域内流动人口政策外生育管理、社会抚养费征收和出生人口性别比综合治理等难题。
　　3.加强省内流动人口服务管理层级监管制度建设和省内区域协调，指导基层认真落实流动人口服务管理职责，将流动人口计划生育工作纳入政府目标管理责任制考核。

　　**四、**组织实施
　　国家人口计生委切实加强全国流动人口计划生育服务管理“一盘棋”工作的组织领导，适时组织开展对各地“一盘棋”工作方案执行情况进行督促检查和评估验收，及时总结通报各地工作进展，将各省（区、市）年度工作完成情况，作为人口计生工作考核评估和奖励的重要依据。国家人口计生委流动人口计划生育工作领导小组各成员单位按照职责做好相关工作，参与相关督查和评估验收。
　　各省（区、市）人口计生委组织领导本省流动人口计划生育服务管理“一盘棋”工作。依据本方案，结合实际制定本省实施方案，加强委内和相关部门的统筹协调，落实各项工作任务。四大协作区牵头省和轮值省负责制定区域年度工作计划，组织协调区域内“一盘棋”工作的落实。西藏自治区人口计生委结合本地流动人口计划生育工作实际，参照本方案执行，并积极创造条件开展工作。
　　附件：1.2012年流动人口计划生育全国“一盘棋”评估标准
　　2.2012年泛长三角区域流动人口“一盘棋”工作计划
　　3.2012年泛珠三角区域流动人口“一盘棋”工作计划
　　4.2012年环渤海区域流动人口“一盘棋”工作计划
　　5.2012年西北区域流动人口“一盘棋”工作计划
　　附件1：
　　2012年流动人口计划生育全国“一盘棋”评估标准

| 项目 | 评估标准 |
| --- | --- |
| 统筹管理 | 1.把流动人口计划生育服务管理纳入地方综合目标考核内容，落实党政“一把手”责任制。  2.流动人口集中的市、县成立流动人口服务管理机构的比例分别达到90%，80%以上；流入人口聚集的乡（镇、街道）有专（兼）职工作人员，流动人口协管人员与流动人口的比例总体达到1：500。  3.建立流动人口服务管理专项经费逐年增长机制；现居住地流动人口人均计划生育经费投入达到户籍人口标准。 |
| 服务均等 | 4.流动人口计划生育免费技术服务落实率达到85%。国家、省级孕前优生检查项目试点地区均将流动人口纳入覆盖人群，并得到较好落实。  5.依法向流动人口提供计划生育优生优育、优先优惠、奖励优待、关怀照顾等服务待遇。  6.依法为流动人口办理一孩生育服务登记，及时办理流动人口信访案件，无因流动人口服务管理问题引发的恶性事件。 |
| 信息共享 | 7.省级流动人口数据库与全员人口数据库实现整合，全员流动人口入库率、流动育龄妇女入库率均达到90%，全员流动人口个案信息准确率达到80%；基层人口计生部门与相关部门建立流动人口信息通报工作机制。  8.完成省级流动人口服务管理信息系统与国家PADIS系统对接；省级系统县级应用率达100%、乡级应用率达到90%。 |
| 区域协作 | 9.重点对象协查信息反馈率达90%、反馈及时率达到90%；网络化协作信息通报接收率均达到80%，接收及时率达到60%。 |
| 双向考核 | 10.完善省、市、县三级层级监管制度，流入、流出地依法履行服务管理职责；利用网络监管信息开展相关工作检查评估。 |

　　注：1.流动人口计划生育免费技术服务落实率＝流入已婚育龄群众免费接受国家规定计划生育技术服务项目的人数/流入已婚育龄群众应当免费接受国家规定计划生育技术服务项目的人数×100%。
　　2.全员流动人口/流动育龄妇女入库率＝符合国家数据上报规范的信息数/上报总数×100%。
　　3.全员流动人口个案信息准确率＝全员流动人口个案信息准确项数/全员流动人口个案信息总项数×100%，前半期指30日有效期的前15日，15日（工作日）有效期的前7日（工作日）。
　　4.重点对象协查信息反馈及时率（网络化协作通报信息接收及时率）＝在协查信息反馈有效期（通报信息接收有效期）前半期已反馈（已接收）的信息数/有效期整期内已反馈（已接收）信息数×100%。
　　附件2：

　　2012年泛长三角区域流动人口“一盘棋”工作计划

　　2012年，泛长三角区域要在国家人口计生委的统一指导和协调下，以科学发展观为统领，积极转变工作思路，不断拓展协作领域和渠道，建立完善“信息互通、服务互补、协商监管、多方共赢”的区域工作新机制，全面深化流动人口计划生育“一盘棋”成果。

　　**一、**召开泛长三角区域协作年度会议，推动区域协作不断深化。适时召开泛长三角区域协作年度会议，总结2011年工作进展，研究区域协作突出问题，交流各省（市）区域协作的做法与经验，进一步深化区域协作。

　　**二、**完善经常性工作制度，促进多层次多领域区域协作。完善区域协作经常性工作制度，区域内各省（市）人口计生部门督促指导本省（市）各级人口计生部门，加强孕环检、社会抚养费征收、“两非”案件查处等方面协作。

　　**三、**强化便民利民举措，维护流动人口计划生育合法权益。依托国家人口计生委PADIS流动人口子系统和各省流动人口信息系统，探索建立居住地与户籍地之间在婚育证明和一孩生育登记等方面双向协作“绿色通道”。

　　**四、**建立交流平台，推进区域协作工作健康发展。搭建区域内流动人口政策交流、工作交流、联络员交流以及基本公共服务均等化试点工作交流平台，交流工作做法与经验，促进区域协作有效开展。

　　**五、**进一步完善泛长三角流动人口计划生育区域协作联络员制度。泛长三角区域协作联络员保持经常性联系，互通信息，加强磋商，协调处理基层在区域协作日常工作中遇到的各类问题。

　　**六、**继续加强信息交换工作，促进区域内信息化建设。依托国家PADIS流动人口子系统和各省流动人口信息系统，积极开展信息交换，组织信息化观摩，共同提升信息化水平。

　　**七、**编印《泛长三角流动人口工作信息》。全年共编印七期《泛长三角流动人口工作信息》，六省一市人口计生委分别编辑一期，顺序和时间安排为：上海（2月）、江苏（4月）、浙江（6月）、安徽（8月）、江西（10月）、湖北（11月）、河南（12月）。
　　牵头省：上海市
　　轮值省：河南省
　　附件3：

　　2012年泛珠三角区域流动人口“一盘棋”工作计划

　　2012年，泛珠三角各省（区、市）将继续加强区域交流和协作，进一步健全流动人口计划生育工作新机制，巩固和深化区域“一盘棋”；坚持以人为本，拓展流动人口计划生育均等化服务范围，维护好流动人口的合法权益。具体工作安排如下：

　　**一、**加强交流沟通，磋商解决重点难点问题
　　继续通过召开联席会议、研讨会等方式，磋商解决政策衔接、信息协查、宣传引领、便民维权、利益导向等领域的问题；积极争取党委、政府领导带队到区域内相关省（区、市）考察学习，交流服务管理经验，商洽区域协作事宜，为基层创造良好的协作环境。

　　**二、**拓展流动人口均等化服务内容
　　加强泛珠各省（区、市）之间在人口和社会发展领域的协同与合作，统筹推进户籍、社会保障、农村土地等相关制度改革，强化职业培训，提升农民工发展能力，推进流动人口市民化，促进社会和谐稳定；争取率先实现流动人口计划生育基本公共服务均等化全覆盖，同时争取党委、政府和各部门的支持与配合，将均等化服务向社会管理层面延伸，促进流动人口社会融合。

　　**三、**尽快出台省际间协作的两项协议
　　在国家人口计生委的指导下，结合泛珠地区工作实际，继续完善《跨省流动人口一孩生育服务登记协作协议》和《流动人口社会抚养费征收协作协议》，争取在区域内尽快实施；督促基层工作人员认真落实相关协议，及时总结研究典型案例，建立严谨、规范的操作流程，形成良好的配合氛围。

　　**四、**加强信息化建设，完善信息共享机制
　　规范源头数据采集，强化信息变更管理，提高数据质量；完善系统对接接口，简化交互流程，做好对接后信息的协查、反馈、接收以及部门间信息共享等工作，及时交换数据，以信息促协作，以信息引导服务上水平。

　　**五、**办好协作专栏
　　办好泛珠协作动态和泛珠协作网络专栏两个栏目，加快信息更新速度，及时共享区域内外成功的服务管理经验，继续编印10期区域协作动态简报，3月-12月每月由一个省编辑一期，编辑顺序依次是：广东、福建、江西、湖南、广西、重庆、四川、贵州、云南、海南。

　　**六、**推动基层协作向务实发展
　　在“双方自愿，共同协商”的原则下，进一步加强打击跨省“两非”行为、合作征收社会抚养费、办理生育服务登记、通报流动人口生育节育信息等方面的沟通和协作；鼓励户籍地在流动人口集中流入的现居住地建立流动人口计划生育协会，成立党支部，利用党支部的优势协助两地人口计生部门开展工作，推进双向服务管理，提高群众自治水平。
　　牵头省：广东省
　　轮值省：海南省
　　附件4：

　　2012年环渤海区域流动人口“一盘棋”工作计划

　　2012年，环渤海地区的流动人口计划生育工作要在全国基本实现“一盘棋”基础上，紧紧围绕巩固完善“统筹管理、服务均等、信息共享、区域协作、双向考核”工作机制，在国家人口计生委流管司领导下，结合环渤海地区流动人口计划生育工作特点，按照区域流动人口工作统筹布局、分步实施、集中研讨、稳步推进的原则，认真贯彻落实《[流动人口计划生育工作条例](https://www.pkulaw.com/chl/f6b2c35d55b7a40ebdfb.html)》和流动人口服务管理“十二五”专项规划，大力推进环渤海区域流动人口计划生育工作不断发展，重点做好以下工作：

　　**一、**召开部署和推进区域“一盘棋”工作联席会议
　　组织召开两次环渤海区域联席会议：
　　1、6月份，在黑龙江省召开环渤海地区“一盘棋”工作会议，请各省主任、分管主任、处长参加。会议主要内容：总结2011年工作，磋商人口发展分布研究、政策衔接、信息协查、宣传引领、便民维权、利益导向等问题。
　　2、9--10月，召开环渤海地区信息化工作推进会议，研究确定2013年工作。请分管主任、处长参加。会议主要内容：交流各省（区、市）区域协作工作情况，进一步深化促进跨区域间协作，为巩固完善全国“一盘棋”打牢基础。

　　**二、**加大推进区域信息化建设力度
　　建设好本省（区、市）的信息系统、交换平台和数据库；努力提高环渤海地区流动人口信息交换平台工作的质量，实现各省（区、市）信息系统一孩生育服务登记、避孕节育情况、违法生育信息在规定的时效内完成提交、核实、确认、反馈工作，提高信息平台的登录率和反馈率，降低“查无此人”的比例。实行区域内流动人口信息定期发布制度，由轮值省（区、市）负责将区域流动人口交换信息向区域各省（区、市）发布，提高各省（区、市）流动人口交换信息的完整性和准确性。

　　**三、**探索建立区域协作制度
　　1、建立联络员工作制度。各省（区、市）流动人口处指派一名工作人员担任联络员，在打击跨省“两非”行为、协助征收社会抚养费、办理跨省一孩生育服务登记、通报流动人口生育和节育信息、依法行政和服务维权等方面进一步加强沟通和协作。联络员通过网络、电话等方式保持经常性的联系，互通信息，处理日常工作事务。
　　2、编印《环渤海流动人口计划生育区域协作动态简报》。由轮值省（区、市）负责收集区域内各省（区、市）流动人口区域协作的最新动态、取得的经验及存在的问题等信息，编辑后印发各省（区、市），同时报送国家人口计生委流动人口服务管理司。各省（区、市）流动人口处联络员将本省每月工作情况及时、主动报送轮值省（区、市）联络员。2012年拟编印2期。简报栏目、内容和版式由轮值省与各省（区、市）协商确定。
　　3、建立环渤海区域协作网站专栏。在各省人口计生委门户网站设立环渤海“一盘棋”专栏，及时报道区域协作的实时动态和国家流动人口服务管理工作的有关精神，共享区域内各省（区、市）的成功经验，公布联席会议达成的协议，借鉴其他区域协作的经验及做法。

　　**四、**编印区域流动人口工作人员通讯录
　　各省（区、市）流动人口处负责收集整理本省（区、市）、市、县、乡各级人口计生部门流动人口管理机构及工作人员联系方式，汇编后将电子文档发送至各省（区、市）自行印制或公布在各省信息系统上。

　　**五、**撰写区域流动人口生存状况报告
　　根据流动人口动态监测和其他相关数据，撰写本省流动人口服务和管理工作调研报告，年底前提供给轮值省人口计生委流动人口服务管理处，编印成册后报国家人口计生委流管司并分发至环渤海各省，为当地经济和社会发展及党政领导决策提供参考。
　　牵头省：北京市
　　轮值省：黑龙江省
　　附件5：

　　2012年西北区域计划生育“一盘棋”工作计划

　　2012年西北区域“一盘棋”工作的总体思路是：以胡锦涛总书记4.26讲话精神为指导，坚持科学发展观，认真贯彻落实国家人口计生委“一盘棋”机制建设一系列指示要求，继续按照西北协作区共同签署的四个管理办法，推进基本公共服务均等化，强化基层基础管理，加快信息化建设，加强保障措施建设，努力提升西北区域“一盘棋”工作水平。重点做好以下几方面工作：

　　**一、**加强对区域内流动人口新情况、新问题的研究
　　按照胡锦涛总书记2011年4月26日在中央政治局第二十八次集体学习时重要讲话精神，把流动人口服务管理上升到加强和创新社会管理的高度，加强对区域内流动人口新情况、新问题的研究。积极探索利益导向衔接、社会抚养费征收、性别比治理等方面的合作。在流动人口计划生育工作协作的基础上，扩大合作范围，提高协作层级。积极探索在现居住地逐步建立和发展“同乡”、行业、企业等流动人口计划生育协会组织，探索依靠流入人口党支部以及“两新”社会组织协助开展计划生育自治工作。

　　**二、**对西北区域“一盘棋”工作进行回顾
　　采取各省专项检查、日常督导与信息化测评、电话调查与基层问卷调查相结合的方式，综合评估西北区域年度流动人口计划生育“一盘棋”工作计划执行情况，主要是“推进均等化服务、强化基础管理、加快信息化建设、加强保障措施”四个协作办法落实情况以及保障机制建设、两地职责落实、开展协作活动、协商重点难点问题等情况。

　　**三、**进一步提高基本公共服务均等化水平
　　一是确保流动人口真正享受到免费的计划生育技术服务，实现计划生育免费技术服务和药具发放全覆盖。确保流动育龄妇女在各自辖区内享受免费的妇女健康检查、婚前检查、孕前筛查。二是实现流动人口育龄群众在各自辖区内享受与户籍人口同等的待遇，与教育、卫生、社保、劳动等部门联合开展便民维权服务，进一步清理各种侵害流动人口权益的行为和规定，严肃查处各类侵权案件，切实维护流动人口合法权益。三是指导各试点城市做好流动人口基本公共服务均等化试点工作，通过率先实现计划生育基本公共服务均等化，带动全社会基本公共服务整体水平的提高。

　　**四、**加强和创新服务管理制度
　　结合各地实际，继续贯彻执行西北区域协议签署的管理工作制度，如流入地“七项”管理制度，流出地“四个一”管理制度。在流动人口聚集较多的县（市、区）和镇（街道、乡）设立流动人口综合服务管理中心，实行“一厅式”服务等。新形势下新生代农民工逐步成为流动人口的主体、流动人口呈现举家迁移和长期化的趋势，对计划生育基本公共服务提出了新的要求，要积极探索新的服务管理模式，加强和创新服务管理制度，为人口流动迁移创造良好的政策和制度环境。

　　**五、**完成信息化建设各项任务
　　2011年西北区域省级流动人口服务管理系统已全部实现与国家系统的对接，2012年重点要提高省级流动人口服务管理信息系统应用水平，提高数据质量，提高信息反馈的及时性和有效性，建立和完善层级监管制度。加大省级系统应用力度，县乡应用率分别达到100%和90%以上，重点服务管理对象信息反馈率达到90%以上，网络化协作避孕节育信息、“一孩生育服务登记”接收率达80%以上。

　　**六、**加强双向管理，强化协作配合
　　在西北地区协作框架下，推进省际间、区域间双向管理和协作，进一步明确细化户籍地和现居住地的双向管理责任。加强毗邻省份协作与省内协作，推动流入、流出两地服务管理职责落实。强化协作配合，通过召开联席会议、协调查办案件、信息通报反馈、协商征收社会抚养费、交流工作经验等，加强省际间重点人群信息核查与反馈，使之经常化、制度化。

　　**七、**继续加强政府保障措施建设
　　一是加强机构队伍建设，积极争取当地党委政府支持，重点加强县乡流动人口服务管理机构建设，按照每5000名现居住人口配备一名社区专职流动人口计划生育协管员的要求，以政府购买公益性岗位的办法配齐配好人员。二是建立经费投入保障机制，按照财政部和国家人口计生委的有关规定，把流动人口服务管理经费按常住人口标准，列入当地财政预算。三是将流动人口服务管理工作纳入党委政府目标责任制考核内容，市、县两级政府每年听取一次流动人口计划生育工作汇报，及时解决存在问题。四是人口计生系统内部的“一把手”要负总责，协调委内资源，形成合力。

　　**八、**组织召开西北区域流动人口计划生育联席会议暨西部地区第十届人口和计划生育协作会
　　按照国家人口计生委确定的西北区域协作区轮值顺序，2012年由宁夏回族自治区担任轮值省。2012年召开西北区域流动人口计划生育联席会议暨西部地区第十届人口和计划生育协作会。
　　牵头省：陕西省
　　轮值省：宁夏自治区

© 北大法宝：（[www.pkulaw.com](https://www.pkulaw.com/)) 专业提供法律信息、法学知识和法律软件领域各类解决方案。北大法宝为您提供丰富的参考资料，正式引用法规条文时请与标准文本核对。
欢迎查看所有[产品和服务](https://www.pkulaw.com/helps/2.html)。法宝快讯：[如何快速找到您需要的检索结果？](https://www.pkulaw.com/helps/70.html)[法宝](https://www.pkulaw.com/helps/69.html)V6有何新特色？

扫描二维码阅读原文

**原文链接：**<https://www.pkulaw.com/chl/240fdbfe97abbc5ebdfb.html>
